# N-terminal-mediated oligomerization of DnaA drives the occupancy-dependent rejuvenation of the protein on the membrane

**DOI:** 10.1042/BSR20150175

**Published:** 2015-09-18

**Authors:** Alexander Aranovich, Shani Braier-Marcovitz, Esti Ansbacher, Rony Granek, Abraham H. Parola, Itzhak Fishov

**Affiliations:** * Department of Life Sciences, Ben-Gurion University of the Negev, P.O.B. 653, Beer-Sheva 8410501, Israel; † Department of Biotechnology Engineering, Ben-Gurion University of the Negev, P.O.B. 653, Beer-Sheva 8410501, Israel; ‡ Department of Chemistry, Ben-Gurion University of the Negev, P.O.B. 653, Beer-Sheva 8410501, Israel; § Department of Biophysical Chemistry, NYU-Shanghai, Pudong, Shanghai 200122, PR China

**Keywords:** bacterial cell cycle, conformation, cross-linking, initiation of deoxyribonucleic acid (DNA) replication, macromolecular crowding, protein-membrane

## Abstract

DnaA, the initiator of chromosome replication in most known eubacteria species, is activated once per cell division cycle. Its overall activity cycle is driven by ATP hydrolysis and ADP–ATP exchange. The latter can be promoted by binding to specific sequences on the chromosome and/or to acidic phospholipids in the membrane. We have previously shown that the transition into an active form (rejuvenation) is strongly co-operative with respect to DnaA membrane occupancy. Only at low membrane occupancy is DnaA reactivation efficiently catalysed by the acidic phospholipids. The present study was aimed at unravelling the molecular mechanism underlying the occupancy-dependent DnaA rejuvenation. We found that truncation of the DnaA N-terminal completely abolishes the co-operative transformation between the high and low occupancy states (I and II respectively) without affecting the membrane binding. The environmentally sensitive fluorophore specifically attached to the N-terminal cysteines of DnaA reported on occupancy-correlated changes in its vicinity. Cross-linking of DnaA with a short homobifunctional reagent revealed that state II of the protein on the membrane corresponds to a distinct oligomeric form of DnaA. The kinetic transition of DnaA on the membrane surface is described in the present study by a generalized 2D condensation phase transition model, confirming the existence of two states of DnaA on the membrane and pointing to the possibility that membrane protein density serves as an on-off switch *in vivo*. We conclude that the DnaA conformation attained at low surface density drives its N-terminal-mediated oligomerization, which is presumably a pre-requisite for facilitated nt exchange.

## Abstract (precis)

Initiation of DNA replication in bacteria requires recharging of DnaA with ATP. We demonstrate in the present study that this process involves the N-terminal domain-mediated oligomerization of the protein on the membrane, which can be modelled as a surface density-driven phase transition switch.

## INTRODUCTION

DnaA is a key protein in one of the central events in the life of the cell, namely, the initiation of chromosome replication, limiting it to once per cell cycle [1]. Its overall activity cycle is driven by ATP binding and hydrolysis that ‘acts as a molecular switch that couples key events during initiation of replication’ [2]. The initiating activity of DnaA is regulated by the type of bound nt and only the ATP–DnaA form is functional [3]. Immediately after replication is initiated, ATP bound to DnaA is efficiently hydrolysed to yield the ADP-bound inactivated form. This negative regulation of initiator function has been termed the regulatory inactivation of DnaA (RIDA) [4,5]. It was shown *in vitro* that at an ATP:ADP ratio of 10 (mimicking that in a live cell) at 37°C, approximately 30 min is needed to spontaneously replace half of the ADP bound to DnaA with ATP. This process is slow due to a very low nt dissociation rate constant [3,6]. Two different DnaA reactivation pathways were proposed to accelerate the nt exchange: (i) interaction with acidic phospholipids, originally described by Kornberg and colleagues ([7–9]; for a recent review, see [10]) and (ii) binding of the protein to specific chromosomal DNA regions termed DARS (DnaA-reactivating sequences) [11]. Both these mechanisms may be involved in DnaA rejuvenation by complementing each other, thus making regulation of the protein more precise and sensitive to subtle physiological changes [12]. In our previous paper, we demonstrated that a small yet physiologically significant part of DnaA is associated with the membrane [13], thus supporting the role of the membrane in regulating DnaA activity.

In the present study, we focused on the acidic phospholipids rejuvenation pathway. We found previously that the rejuvenation of DnaA by the membrane depends on the protein surface density, rather than initiated by binding itself [6]. Namely, two states of DnaA on the membrane were observed in the presence of acidic phospholipid liposomes: with low and high nt dissociation rate constants, states I and II respectively. The transition between these states is co-operative with regard to the protein density on the membrane, i.e. membrane occupancy [6]. The strong dependence of this phenomenon on the protein surface density implies that one of the states corresponds to a specific DnaA oligomeric form promoted by macromolecular crowding [14,15]. Structural–functional analysis demonstrates that initiation activity of DnaA is accompanied by its oligomerization on the origin of chromosome. Initiation begins with the formation of the DnaA complex on *oriC* and then proceeds with the unwinding of adjacent AT-rich regions. The complex is composed of ∼20 molecules of DnaA, as estimated from EM [16] and confirmed by the surface plasmon resonance technique [17]. There are at least two sites of DnaA–DnaA interaction. One of them is found on the N-terminal of DnaA. Residues 1–86 were shown to be sufficient for DnaA oligomerization *in vitro* [18], which was confirmed by subsequent studies [19–21]. Another potential DnaA–DnaA interaction site is found on domain III of DnaA, which is responsible for ADP/ATP binding. This domain belongs to the ATPases associated with a variety of cellular activities (AAA+) [22,23], which consists of proteins bearing a wide variety of functions. AAA+ ATPases are known to form primarily hexameric rings [24], although heteropentameric [25,26] and heptameric rings [27] have been observed as well. In addition, it was demonstrated that the box VII motif of *Escherichia coli* DnaA protein is required for oligomerization [28]. According to its crystal structure, oligomerization of DnaA in solution is nt dependent: whereas the initiation-inactive ADP-form of DnaA is crystallized as a monomer, the active ATP-form is crystallized as a quaternary structure organized into a right-handed superhelix multimer [22]. Finally, oligomerization of DnaA on a DNA substrate was demonstrated by site-specific cross-linking [29]. However, the possibility that DnaA may form oligomers on the membrane was never tested.

To unravel the molecular mechanism underlying the membrane-induced kinetic transition of DnaA, we examined how the DnaA N-terminal is involved in the transformation process from state I to II and in the oligomerization of DnaA on the membrane at varying protein surface densities. For this purpose we used N-terminal truncated DnaA (tDnaA) and an environmentally-sensitive fluorescent probe 2-(4-maleimidoanilino)naphthalene-6-sulfonic acid (MIANS), specifically attached to the N-terminal of DnaA. The direct detection of DnaA–DnaA interaction and the resulting oligomerization at varying phospholipid-to-protein ratios (PL:Pr) was achieved by chemical cross-linking. We suggest a phase transition model for description of DnaA kinetic states on the membrane and provide a scheme summarizing the proposed membrane-driven mechanism of DnaA rejuvenation.

## EXPERIMENTAL

### Materials

1-Stearoyl-2-oleoyl-*sn*-glycero-3-[phospho-*rac*-(1-glycerol)] (SOPG) was obtained from Avanti Polar Lipids, MANT-ATP was obtained from Molecular Probes and Ni–NTA (nitrilotriacetic acid) was obtained from Qiagen; 1.2-bis-maleimidoethane (BMOE) was obtained from Pierce.

### Preparation of N-terminal truncated DnaA mutant

N-terminal tDnaA was prepared by using a primer located inside the *dnaA* gene with the NdeI restriction site, 5′-gcgc-cttctacgcgctcaCATATGgataacgtcccggccccggc-3′ and the 3′-terminal complementary primer 5′-ggcagcagccaactcagcttcctt-tcggg-3′ located inside the vector. The obtained PCR product was 348 bp shorter than the original *dnaA* gene from the 5′-terminal of the gene. The final PCR product of the mutated gene was digested with NdeI (BioLabs) and BamHI (MBI) and then inserted into the pET19b (Novagen) vector and subsequently transformed into *E. coli* strain BLR(DE3)pLysS (Novagen). The molecular mass of His-tagged tDnaA was determined by MALDI–MS and appeared to be 42.778 Da, 273 Da (0.6%) larger compared with a predicted mass. The difference is, most probably, due to the accuracy of the technique.

### Protein purification and preparation of liposomes

Recombinant polyhistidine-tagged DnaA and tDnaA proteins were purified on Ni–NTA beads (QIAGEN) as described previously [6]. Protein concentration was measured by Bradford's method [30] using BSA as the standard. The obtained concentrations were found to be comparable with measurements of native protein absorption at 280 nm. Protein purity and size were estimated by SDS/PAGE stained with Coomassie Brilliant Blue [31]. Liposomes composed of SOPG were prepared by an extrusion method [6].

### Fluorescence measurements

All fluorescence data were acquired on PerkinElmer Fluorometer LS-55 (PerkinElmer). Fluorescence spectra were collected with 0.5 nm steps. For kinetic measurements fluorescence intensity of 3′-*O*-(*N*-methylanthraniloyl) [MANT]–ATP was acquired every 1 s at fixed wavelengths *λ*_ex_=352 nm and *λ*_em_=442 nm (for details see [6]). Fluorescence intensity of MIANS was measured at *λ*_ex_=327 nm and *λ*_em_=430 nm. Vertical and horizontal polarizers were introduced for excitation and emission respectively, to reduce the contribution of scattered light in the presence of liposomes without affecting relative changes in fluorescence intensity.

### Membrane-dependent dissociation kinetics of MANT–ATP

MANT–ATP is a fluorescent analogue of ATP. In spite of its 4-fold reduced affinity to DnaA relative to that of ATP, it was shown to be suitable for studying nt–DnaA binding kinetics [6]. All the kinetic measurements followed the procedures described previously [6].

### DnaA labelling with MIANS

ATP–DnaA (25 μM) was incubated with 50 μM of MIANS (also called Mal-ANS), in 220 μl of buffer HD (50 mM PIPES-KOH (pH 6.8), 10 mM magnesium acetate, 20% (w/v) sucrose, 200 mM ammonium sulphate) without DTT for an hour at room temperature. The reaction was stopped with 1 mM of DTT and desalted in two subsequent size exclusion columns, each filled with 1 ml of Sephadex G-25 equilibrated in buffer AC (50 mM PIPES-KOH (pH 7), 30 mM KCl, 2.5 mM magnesium acetate, 20% (w/v) sucrose, 0.1 mM EDTA, 2 mM dithiothreitol) containing 1 mM ATP. The samples were eluted by centrifugation (2000 ***g*** for 1.5 min). Final protein and MIANS concentrations were determined by Bradford's method and the absorption at 327 nm (*ε*=27,000 M^−1^cm^−1^) respectively. The calculated DnaA:MIANS ratio was ∼1. The association kinetics of MIANS with DnaA was fitted with the double-exponential rise to a maximum equation.

### Partial digestion of MIANS–DnaA

ATP–DnaA was reacted with MIANS exactly as described in the previous section and after stopping the reaction with DTT the mixture was centrifuged for 2 min at 16000 ***g*** instead of desalting. Next, the supernatant was incubated in 70% hydroxylamine buffer [400 mM CHES-NaOH (N-cyclohexyl-2-aminoethanesulfonic acid; pH 9.7), 2.7 M hydroxylamine-hydrochloride] for 16 h at 37°C [32]. The sample was centrifuged for 5 min at 16 000 ***g*** rcf and 20 μl of bottom fraction was separated on SDS/PAGE (18% gel). The fluorescence of MIANS was visualized under UV light and then stained with Coomassie Brilliant Blue. The size of the DnaA fragments was determined by a protein size marker that includes two UV excited bands, 25 and 75 kDa [Precision Plus Protein™ Standards, Dual Color (Bio-Rad)].

### Environmental changes in the MIANS-labelled N-terminal of DnaA with increasing membrane occupancy

The experiment consisted of two types of titrations in the absence or presence of 30 μM of SOPG liposomes with MIANS–DnaA. The relative increase in intensity of MIANS–DnaA fluorescence, upon binding to the membrane, was calculated as follows:

Fluorescence intensity of one molecule of MIANS–DnaA in solution is (*F*^0^_S_):

1 $$F_{S}^{0}=\frac{F_{S}}{P_{T}}$$

where *F*_S_ is the measured fluorescent intensity signal in the absence of membranes and *P*_T_ is the total MIANS–DnaA concentration.

The fluorescence intensity of one molecule of MIANS–DnaA bound to the membrane is (*F*^0^_M_):

2 $$F_{M}^{0}=\frac{F_{M}-F_{S}^{0}\cdot \left(P_{T}-P_{B}\right)}{P_{B}}$$

where *F*_M_ is the measured fluorescent intensity signal in the presence of membranes and *P*_B_ is the membrane-bound MIANS–DnaA calculated as in Aranovich et al. [6]. The relative fluorescence intensity increase is therefore:

3 $$\frac{F_{M}^{0}}{F_{S}^{0}}=\frac{F_{M}-F_{S}^{0}\cdot \left(P_{T}-P_{B}\right)}{F_{S}^{0}\cdot P_{B}}$$

### Cross-linking

Oligomerization of DnaA under various conditions was detected directly by cross-linking. For this purpose, the homobifunctional thiol-reactive reagent BMOE was used. The procedure was as follows: pre-centrifuged (300000 ***g***, 20 min, 25°C) ATP–DnaA (0.8 μM final concentration) was added to 150 μl of reaction mixture containing buffer AC (without DTT), 1 mM ATP, 2.4 μM BMOE in the presence or absence of SOPG (1 μl of stock solution of BMOE in DMSO was added, which corresponds to 0.7% of DMSO in the reaction mixture). The mixture was incubated for 20 min at 30°C and the reaction was stopped by adding 2.5 mM of DTT. After the sample buffer was added, 40 μl of the sample was run on non-reducing SDS/PAGE (10% gel) and the DnaA oligomeric forms were visualized by Coomassie Brilliant Blue staining.

All gel densitometry analyses were performed with ImageJ.

## RESULTS

### Characterization of N-terminal truncated DnaA(Δ1–117)

**Figure 1:**
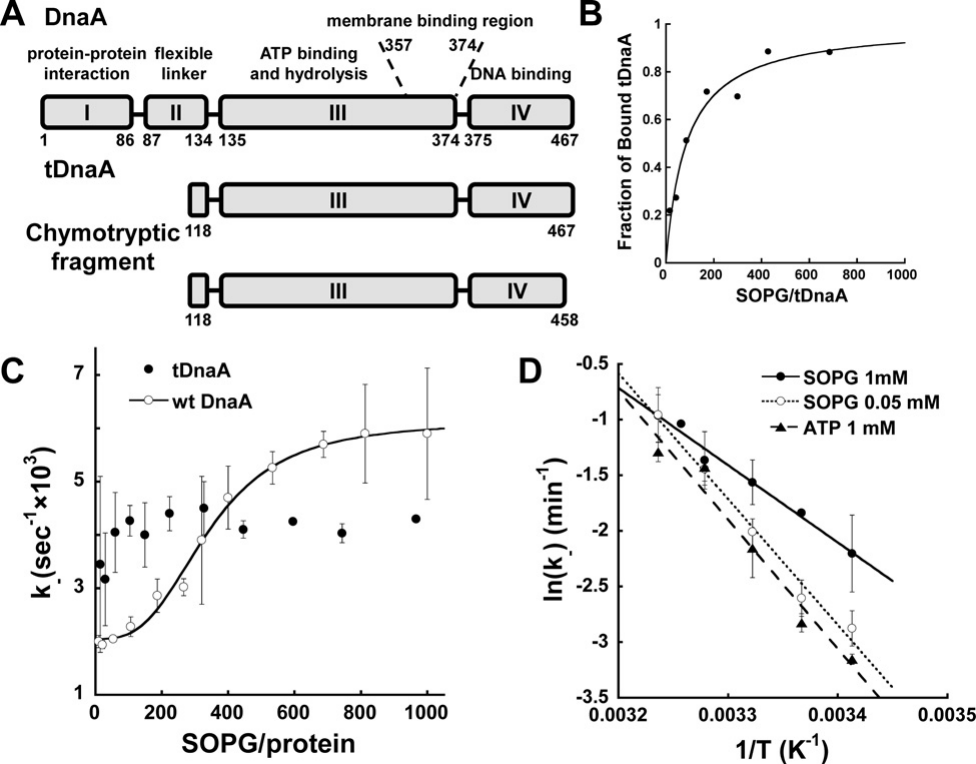
The DnaA N-terminal is essential for the formation of state II on the membrane (**A**) Schematic representation of the functional domains of the *E. coli* wtDnaA protein with the membrane-binding amphipathic helix indicated. (**B**) Binding of ATP–tDnaA (0.73 μM) to the SOPG (5–500 μM) liposomes determined by co-sedimentation [6]. The relative quantity of sedimented tDnaA was calculated, then normalized to maximal binding and plotted against the PL:Pr ratio. Data were fitted with a standard binding isotherm (*R*^2^=0.98). (**C**) Dissociation kinetics of the MANT–ATP·DnaA complex on the PL:Pr ratio. The dissociation was induced by the addition of SOPG (10–1000 μM) to 0.8 μM of wtDnaA (○) or tDnaA (●) pre-incubated with MANT–ATP (0.045 μM). The rate constant values were calculated from single exponential decay fits (Aranovich et al. [6]). The data are the average of 3–5 independent experiments for each point. (**D**) Arrhenius plot of temperature dependences of the dissociation rate constant for the MANT–ATP·tDnaA complex. Kinetics were measured in a 20°C–36°C temperature range; dissociation was induced by low (○) or high (●) liposome concentrations (50 μM or 1 mM SOPG respectively). The data were fitted with linear regression (*R*_0_^2^ > 0.95).

In view of the role of the N-terminal domain in DnaA oligomerization on *oriC* [18–21], we attempted to explore the role of this domain in DnaA occupancy-mediated transformation from the inactive state I to the active state II on the membrane. For this purpose, N-terminal tDnaA was prepared by genetic engineering techniques (Experimental). The tDnaA(Δ1–117) was designed based on literature data, demonstrating that the chymotryptic fragment of DnaA, i.e., amino acids 118–458, including domain III and most of domain IV, is able to bind nts and DNA and that it retains its sensitivity to acidic phospholipids as well (Figure 1A) [33].

The tDnaA characteristics were tested in comparison with full-length DnaA (wtDnaA) protein for its membrane binding and rejuvenation abilities (Figures 1B–1D). The affinity of the mutant protein to liposomes composed of acidic phospholipids was assessed using the co-sedimentation technique (Figure 1B) [6]. Fitting the results with a standard binding isotherm leads to a dissociation constant for SOPG liposomes, *K*_D_(SOPG), of 62.8±18.9 μM close to that measured for wtDnaA, *K*_D_(SOPG)=43.4±15.0 μM [6]. The tDnaA protein binds the fluorescent analogue of ATP, MANT–ATP and releases the nt in the presence of membranes or an excess of unlabelled ATP (Figures 1C and 1D). It can be concluded that tDnaA retains the features of membrane and nt binding, which can be compared with those of wtDnaA relating to membrane occupancy. This is consistent with our previous finding that the 117–378 fragment of DnaA preserves full membrane retention capability *in vivo* [13].

The dissociation of the MANT–ATP–DnaA complex is a rate-limiting step in the nt exchange and its rate can be used as to indicate the efficiency of DnaA rejuvenation by the membrane. This conclusion is based on the following findings: (i) the rate of MANT–ATP binding to wtDnaA is faster than its dissociation [6]; (ii) the binding rate of wtDnaA to the membrane is much faster (beyond the temporal resolution of our measurements) than the rate of nt dissociation, as evident from the FRET between fluorescently labelled wtDnaA and membrane-embedded probe [34], changes in the membrane dynamics [6], as well as changes in MIANS–wtDnaA fluorescence intensity (see below).

The dissociation kinetics of the MANT–ATP·tDnaA complex at various SOPG concentrations was compared with that of MANT–ATP·wtDnaA (Figure 1C). Each data point on the graph was obtained from a first-order exponential decay fit of the dissociation kinetics (Figure 4) [6]. Figure 1(C) shows that MANT–ATP·tDnaA dissociation exhibits independence regarding the PL:Pr ratio in contrast with the co-operative transformation observed for wtDnaA. In addition, the observed rate constants for MANT–ATP·tDnaA have values intermediate to those observed for the two states of wtDnaA. This indicates that the N-terminal of wtDnaA is essential for forming either of the protein states (I or II) on the membrane. To identify which of the states (or both) of tDnaA has been lost due to the absence of the N-terminal relative to wtDnaA, we measured the temperature dependencies of the dissociation rate constant of the MANT–ATP·wtDnaA complex at high and low membrane occupancies (Figure 1D). The results are summarized in Table 1. It is evident that at high protein density on the membrane surface (corresponding to state I of wtDnaA) and in the aqueous phase, the thermodynamic characteristics of the dissociation of the tDnaA and wtDnaA nt complexes are similar, thus indicating that they are in the same protein state. However, at low membrane occupancy, ‘state II’ of tDnaA is much different either from that of the wild-type state II or from that at high membrane occupancy (state I). Moreover, at physiological temperatures binding of tDnaA to the membrane promotes nt release to the same extent as the excess of ATP, in contrast with the substantial acceleration in the case of wtDnaA (compare with Figure 5C in [6]). This result indicates that the N-terminal of the protein is required for forming the wtDnaA state II at low membrane occupancy.

**Table 1:** Comparison between wtDnaA and tDnaA based on the measurements of the dissociation rate constant at different temperatures and at different SOPG:protein ratios (Figures 1C and 1D)

		Dependence of the dissociation rate constant on temperature*		Dissociation rate constant behaviour in the aqueous phase and at varying SOPG:protein ratios		
Protein type	Occupancy	*E*_A_, kcal/mol	ln(A), min^−1^	k^−^, × 10^3^ s^−1^ at 30°C	*K*_A_^†^	Hill coefficient (*n*)^†^
wtDnaA	High, State I	23.8±1.1	37.2±1.8	2.05±0.09	343±18	3.02±0.46
	Low, State II	36.3±1.1	58.7±1.8	6.13±0.24		
	Aqueous, ATP	23.7±1.2	37.0±2.1	2.50±0.20		
tDnaA	High	22.6±1.5	35.6±2.6	3.97±0.37	-	-
	Low	13.9±0.9	21.5±1.5	4.57±1.27		
	Aqueous, ATP	23.2±2.5	36.4±4.2	4.05±0.65		

*Activation energies and pre-exponential factors calculated from Arrhenius equation fits (for all fits *R* ≥ 0.99, the data are summarized for 2–3 independent experiments [6].

†*K*_A_=activation constant, phospholipid/protein mole ratio at which the nt dissociation rate constant is half-maximal, as calculated from the Hill equation fit.

### Environmental changes in the MIANS-labelled N-terminal of DnaA at varying membrane occupancies

**Figure 2:**
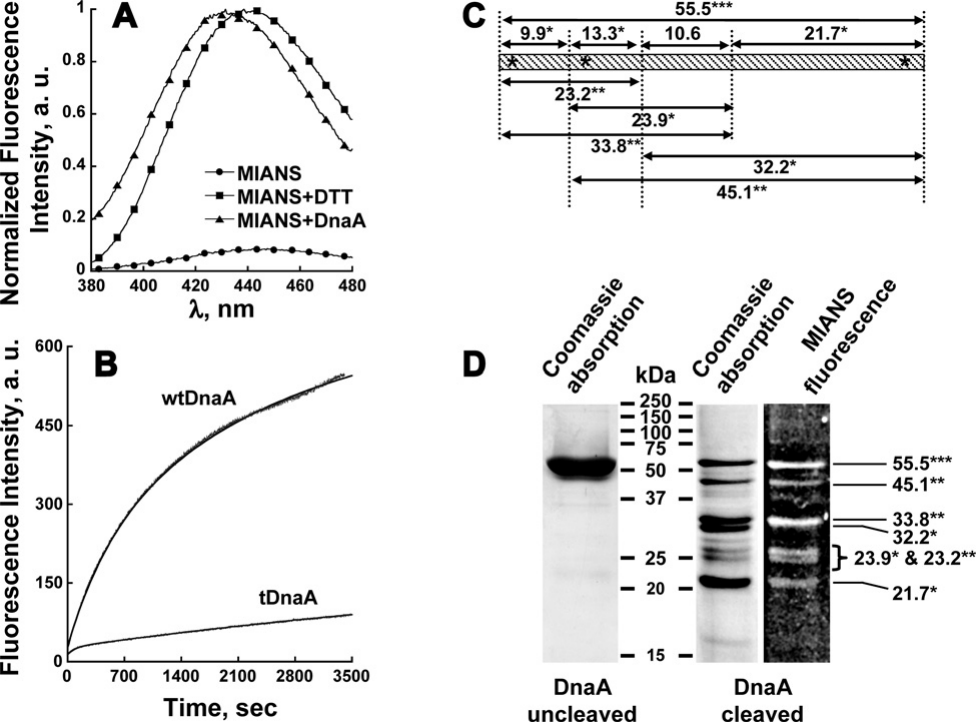
Specific labelling of DnaA N-terminal by MIANS (**A**) Fluorescence spectral characteristics of MIANS. Fluorescence intensity of 2 μM of free MIANS (●) increased more than 10-fold upon binding to DTT (■) or DnaA (▲). The representative spectra of MIANS–DTT and MIANS–DnaA were normalized to the intensities at their emission spectral peaks respectively. A fluorescence spectrum of free MIANS was normalized to the peak of MIANS–DTT. (**B**) Binding kinetics of MIANS to wtDnaA and tDnaA. The reaction begins after the addition of 4 μM MIANS to buffer HD containing 1 mM of ATP and 0.8 μM of ATP–wtDnaA or ATP–tDnaA without DTT. Excitation and emission wavelengths were 327 and 430 nm respectively, with 3 nm slits. The experiment was performed at room temperature. The data were fitted to a double-exponential rise to the maximum equation (*R*^2^ ≥ 0.999). The results are summarized in Table 2. (**C** and **D**) Partial hydroxylamine cleavage of DnaA. (**C**) Hydroxylamine cleavage map of His-tagged wtDnaA. The map summarizes all possible theoretical fragments expected from a partial protein digestion with hydroxylamine. (**D**) ATP–DnaA was pre-incubated with a 2 molar excess of MIANS and separated on SDS/PAGE (18% gel). The fluorescence of MIANS was visualized under UV-light and then the gel was stained with Coomassie Brilliant Blue (Experimental). Asterisks indicate the number of cysteines in a protein fragment.

Based on MANT—ATP–tDnaA kinetics (Figures 1C and 1D; Table 1), together with the previously reported results [6], it can be concluded that the N-terminal of DnaA plays a crucial role in the co-operative transition of DnaA between the two functional states with low (state I) and high (state II) nt dissociation rates. Presumably, this transition is accompanied by specific local environmental changes in the protein due to either its oligomeric state, conformational changes or both. Since the lack of the DnaA N-terminal eliminates this co-operative transition, it is very likely that this specific change takes place at the N-terminal of the protein. Accordingly, we utilized an environmentally-sensitive fluorophore, MIANS, which has been widely used for protein structural studies [35]. MIANS binds specifically to cysteine moieties and is not fluorescent until its maleimide group reacts with the thiol moiety, e.g. cysteine or DTT [36]. The fluorescence spectral characteristics of free MIANS and MIANS-labelled DnaA are shown in Figure 2(A). As expected, the fluorescence intensity of MIANS increased more than 10-fold upon binding to DTT with a spectral blue-shift of 9 nm, from 447 to 438 nm (Figure 2A). An even greater blue-shift of 17 nm, 447 to 430 nm, was observed for MIANS attached to DnaA (Figure 2A), indicating that the fluorophore bound to the protein is situated in a more hydrophobic environment compared with the aqueously exposed DTT–MIANS.

**Table 2:** Kinetics parameters of MIANS association with wtDnaA and tDnaA (Figure 2B)

	Reaction component	*F*, a.u.	Relative Signal*	*k*_+_, s^−1^ × 10^3^
wtDnaA	fast	180±7	0.28	1.97±0.05
	slow	455±2	0.71	0.38±0.01
tDnaA^†^	slow	184±1	0.29	0.12±0.00

*The relative signal was calculated as the ratio of the maximal fluorescence intensity signal of MIANS bound to the protein obtained from the fit (*F*, a.u.) divided by the overall maximal signal of wtDnaA.

†The binding kinetics of MIANS to tDnaA fits well with a single-exponential equation. The contribution of the second exponent is neglected.

DnaA contains three cysteines, Cys^9^, Cys^67^ and Cys^441^, enabling targeted thiol-specific protein labelling. Cys^9^ and Cys^67^ are located on the DnaA N-terminal, whereas Cys^441^ is situated on the C-terminal of the protein. In order to specifically label the DnaA N-terminal, we exploited the accessibility of the cysteine moieties to MIANS. The accessibility was determined by two different types of experiments. The first one measures the binding kinetics of MIANS to wtDnaA (three cysteines) in comparison with tDnaA containing only Cys^441^ (Figure 2B). In order to label all DnaA cysteines, a 5 molar excess of MIANS was added to the protein and the increase in fluorescence intensity was followed. Analysis of the fluorescence kinetics (Table 2) revealed that (i) wtDnaA binds MIANS at approximately 3-fold greater quantity than does tDnaA, presumably reflecting the cysteine content and the resulting stoichiometric ratio in each. (ii) The binding kinetics of MIANS to wtDnaA may be fitted into a two-exponential rise, fast (approximately one-third of the total fluorescence change) and slow (approximately two-thirds of it). It differs by approximately one order of magnitude, whereas the tDnaA-binding kinetics is composed of only one component. Since tDnaA exhibits slow binding kinetics, it can be concluded that the fast component of the binding kinetics corresponds to the N-terminal cysteines.

Selectivity of labelling was achieved by lowering the probe concentration, limiting the incubation time of DnaA with MIANS and reducing the reaction temperature. The above difference in binding kinetics was utilized for selective labelling of wtDnaA, with a 2:1 ratio of MIANS per protein and a relatively short, 1 h incubation time at room temperature (see Experimental). Under these conditions, only the wtDnaA-N-terminal is expected to be labelled. Partial digestion of wtDnaA with hydroxylamine (NH_2_OH) verified the MIANS labelling sites. Hydroxylamine cleaves asparaginyl-glycine protein peptide bonds with high selectivity [32]. An important advantage of using hydroxylamine is that it can digest the protein under denatured conditions, thus overcoming accessibility barriers in contrast with protease cleavage. The wtDnaA fragment map (Figure 2C) summarizes all possible theoretical fragments expected from partial protein digestion with hydroxylamine. It can be concluded that the hydroxylamine digestion profile enables one to separate and identify N-terminal cysteines from Cys^441^. Next, MIANS-labelled ATP–wtDnaA was exposed to hydroxylamine and the fragments obtained were separated on SDS/PAGE gel (Figure 2D). The total intensity of each fragment for Coomassie staining (*I*_protein_) and MIANS fluorescence (*I*_MIANS_) was determined. The efficiency of labelling was calculated as the (*I*_MIANS_):(*I*_protein_) ratio and is summarized in Table 3. Fragments containing Cys^441^ alone, 32.2 and 21.7 kDa were poorly stained with MIANS. This result indicates that under the chosen conditions, Cys^441^ remains generally unlabelled, thus confirming the kinetics results (Figure 2B). It is difficult to distinguish between the reactivities of the two N-terminal Cys^9^ and Cys^67^; however, the overall labelling stoichiometry obtained under the labelling conditions used was 1:1, thus suggesting that either one of them was labelled in each protein molecule.

**Table 3:** Labelling efficiency of wtDnaA cysteines by MIANS

Peptide, kDa	Cysteine content, residue number	*I*_MIANS_:*I*_protein_*
55.5	9, 67, 441	7.6
45.1	67, 441	4.1
33.8	9, 67	3.4
32.2	441	0
23.9 and 23.2	67 and 9, 67	6.2 or 2.7
21.7	441	0.6

*Fragments of MIANS-labelled ATP–wtDnaA treated with hydroxylamine were separated on SDS/PAGE gel (Figure 2D) and the bands analysed using ImageJ (see the text for explanations).

The MIANS-labelled protein retained its activity. This was evident from the unchanged binding/release of MANT–ATP from N-ethylmaleimide (NEM)-modified protein by excess ATP or by adding phospholipids, both at a high or at a low PL:Pr ratio (result not shown). NEM-labelled protein was utilized for this purpose in order to avoid the fluorescence overlap between MIANS and MANT. The identity between the reactive moieties in NEM and MIANS enabled us to validate it.

**Figure 3:**
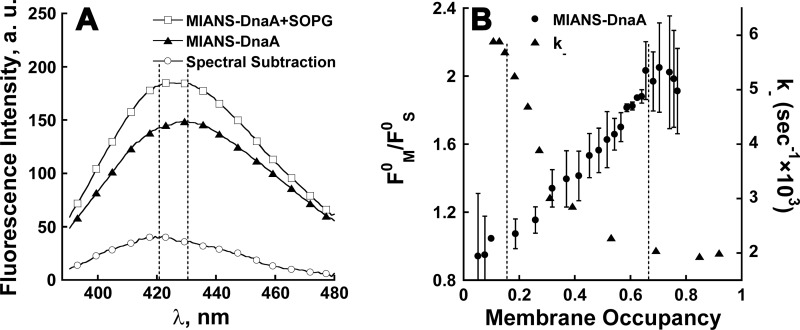
Environmental changes in MIANS–DnaA correlate with changes in the nt dissociation rate constant at varying membrane occupancies (**A**) MIANS is a good sensor for DnaA conformational changes in the membrane. (A) Representative emission spectra of 0.4 μM MIANS–DnaA in the absence (▲) or the presence of 30 μM SOPG (□) and their resulting spectral subtraction (○). (**B**) Environmental changes in MIANS–DnaA at varying membrane occupancies. Buffer AC containing 1 mM ATP in the absence or the presence of 30 μM of SOPG liposomes was titrated with MIANS–DnaA (0.007–0.846 μM) at 30°C (two of four independent experiments). The relative fluorescence intensity increase in membrane-bound MIANS–DnaA (*F*^0^_M_/*F*^0^_S_) was calculated using eqns (1)–(3) and plotted against the calculated membrane occupancy (●). The result was compared with the dissociation kinetics of the MANT–ATP·DnaA complex at varying PL:Pr ratios (Figure 1C; [6]) presented in the same occupancy coordinates (▲). The measurement conditions were as follows: 600 μl of buffer AC, 30°C, excitation and emission slits 10 nm, excitation wavelength 327 nm, emission at a fixed wavelength of 430 nm.

Further experiments demonstrated that MIANS is an effective fluorescent sensor for detecting density-dependent environmental changes in DnaA on the membrane (Figure 3). At high membrane occupancy, when only ∼40% of the protein is bound, the total fluorescence intensity of MIANS–DnaA increases along with a blue-shift of 3 nm (Figure 3A). The calculation of membrane-bound protein is based on co-sedimentation results (Figure 1B) [6]. It can be concluded that (i) the blue-shift is expected to be markedly higher than 3 nm but less than 9 nm, which comes from spectral subtraction and (ii) the intensity of membrane-bound MIANS–DnaA increases ∼2-fold upon binding to the membrane at high occupancy (Figure 3A). Indeed, the titration of SOPG liposomes with MIANS–DnaA resulted in up to a 2.2-fold fluorescence intensity increase in the membrane-bound labelled protein at high occupancy (Figure 3B). This increase does not result from just protein binding to the membrane, because at low membrane occupancy, when most of the protein is bound to the membrane, no increase in MIANS–DnaA fluorescence intensity was observed (Figure 3B). Rather, the changes correlate with the protein density on the membrane. Notably, the transition range of the relative fluorescence intensity of MIANS-wtDnaA is between 0.15 and 0.7 membrane occupancy, which actually coincides with that of the dissociation rate constant of the MANT–ATP.wtDnaA complex (Figures 1C and Figure 3B) [6]. The latter suggests that the transformation between states I and II of wtDnaA is associated with changes in the MIANS environment at its labelling site on the N-terminal of wtDnaA.

### Oligomeric forms of DnaA on the membrane are revealed by cross-linking

Several previous reports show that residues 1–86 of N-terminal of DnaA are sufficient for DnaA oligomerization [18–21]. Together with the above results indicating conformational changes in the protein on the membrane (Figure 3), it appears highly likely that the kinetically manifested states I and II (Figure 1) [6] originate from different oligomerization states of the protein on the membrane. In order to measure directly the degree of oligomerization and the oligomeric forms of DnaA at various protein densities on the membrane surface, we used the cross-linking technique. For this purpose, we chose the homobifunctional thiol reagent bismaleimidoethane (BMOE), which has been used elsewhere to characterize DnaA oligomerization [29]. Owing to the short [8 Å (1 Å=0.1 nm)] and rigid spacer arm, BMOE is expected to provide selective cross-linking between closely adjacent cysteine moieties. Moreover, since BMOE has the same maleimide reactive group as MIANS, a similar reactivity with the three DnaA thiol groups is expected. The presence of two readily reactive cysteines on the N-terminal (Figure 2) makes possible the formation of not only dimers, but also trimers and higher order oligomers as a result of cross-linking.

**Scheme 1:**
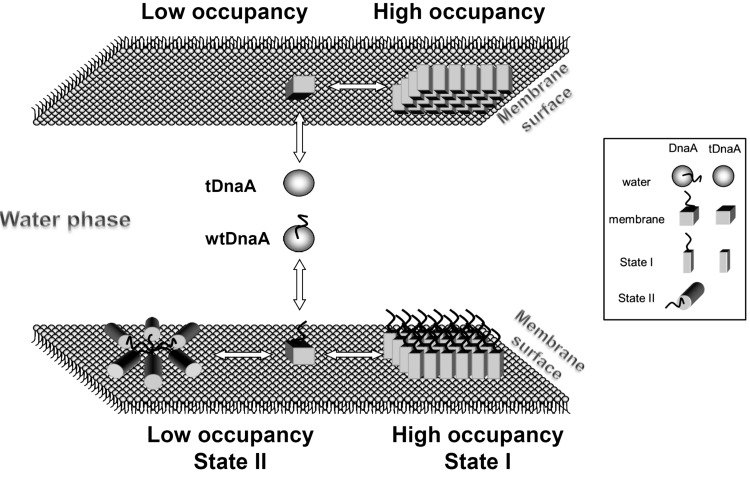
DnaA transformation model Proposed conformational states and the resulting oligomeric forms of wild-type (bottom) and N-terminal truncated (top) DnaA in aqueous phase and different membrane occupancies (protein surface densities). The shapes (sphere, cube, prism and cylinder) symbolize the kinetically distinguishable forms of DnaA and tDnaA and their corresponding conformations as designated. Only the accessible monolayer of the membrane is shown for simplicity. See the text for more explanations.

**Figure 4:**
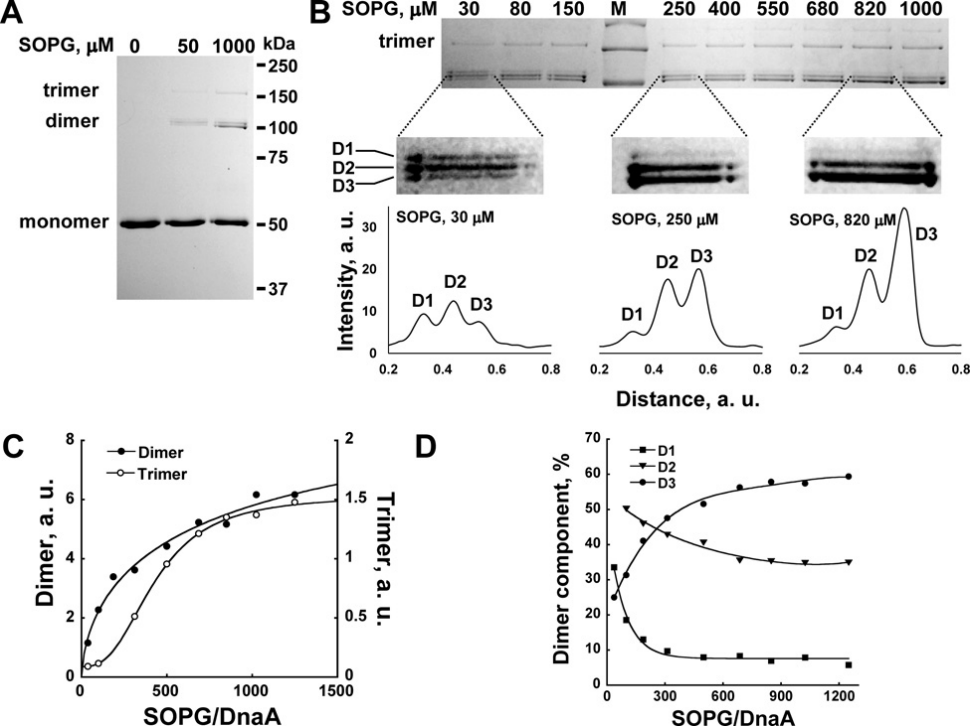
Two distinct types of oligomers at high (state I) and low (state II) membrane occupancy revealed by cross-linking (**A** and **B**) Cross-linking of DnaA in the presence of varying SOPG concentrations. DnaA (0.8 μM) in the presence of varying SOPG concentrations was cross-linked with 2.4 μM homobifunctional thiol reagent BMOE in buffer AC without DTT at 30ºC (see Experimental). The samples were separated on acrylamide non-reducing SDS/PAGE (10% gel) and visualized by Coomassie Brilliant Blue staining. (**A**) DnaA cross-linking of in aqueous phase (0 μM), low (50 μM) and high (1000 μM) SOPG concentrations. These are representative results from numerous repeats. (**B**) DnaA was cross-linked at varying SOPG concentrations (30–1000 μM) and separated on non-reducing SDS/PAGE as in (**A**). The plot profiles of the DnaA dimers are shown at the bottom for 30, 250 and 820 μM of SOPG. (**C** and **D**) The total amount of dimers and trimers increases at higher SOPG:DnaA ratios. The dimers (●) and trimers (○) on the gel image (**B**) were analysed by densitometry using the ImageJ program and the obtained data were plotted against SOPG:DnaA. (**C**) The dimers’ and trimers' data were fitted to the Hill equation with *R*^2^=0.98 and *R*^2^=0.999 respectively. (**D**) Different behaviour of three types of detectable dimers. D1 (■) and D2 (▼) decrease whereas D3 (●) increases at higher SOPG:DnaA ratios. The lines are only to guide the eye.

Indeed, dimers, trimers and even higher order oligomers of wtDnaA were detected in the presence of SOPG liposomes, but not in their absence (Figure 4A). Up to 20% of wtDnaA was found to be in dimeric and trimeric forms at high SOPG concentrations at BMOE:DnaA molar ratios of 3 after 20 min incubation with the cross-linker at 30°C. Notably, the yield of cross-linked products did not significantly increase at higher BMOE:DnaA ratios, up to 20 or upon longer incubation times (result not shown). Cross-linking of wtDnaA with increasing SOPG concentrations was performed, clearly showing that the total amount of oligomers increases with an increase in the SOPG:DnaA ratio (Figure 4B). The amounts of dimers and trimers were quantified by densitometry and the data obtained were plotted against SOPG:DnaA and fitted to the Hill equation (Figure 4C). Co-operativity was negative for the formation of DnaA dimers and positive for trimers with Hill coefficients of 0.62±0.16 and 2.15±0.33 respectively. The mid-point value of SOPG:DnaA for trimers is 428±35 (Figure 4C), which resembles that obtained under the same conditions for the dissociation rate constant of the MANT–ATP–DnaA complex (343±18; Figure 1C). Moreover, three types of dimers were observed, D1–3, whose quantities depend differently on the protein density on the membrane (Figure 4B). The fractions of D1 and D2 decrease and the fraction of D3 increases with decreased membrane occupancy (Figure 4D), along with an overall increase in the oligomer quantity (Figure 4C). The existence of distinctly separated dimers is, most probably, the result of different inter-cross-linked pairs of cysteines. Assuming that only the two N-terminal cysteines are involved, there are three possible single and two double inter-cross-linked products that could run differently on SDS/PAGE due to different degrees of compactness. The formation of each specific inter-cross-linked pair is apparently dependent upon the distances between the cysteines in the pair prior to cross-linking. The distance may change due to changes in the orientation of the DnaA molecules relative to each other and/or the conformational changes in the protein. Since the total amount of cross-linked products increases with a decrease in protein density on the membrane (Figure 4C), we conclude that DnaA undergoes oligomerization rather than just conformational changes at state II of the protein. Overall, the results suggest that the D1 and D2 dimers reflect the DnaA oligomeric form at high protein concentrations on the membrane (state I), whereas D3 and trimers reflect a distinct form of oligomers specific to the low protein density on the membrane (state II) (see Scheme 1).

### Phase transition model for DnaA kinetic states on the membrane

We have previously ascribed the reported sigmoidal shape of the reaction rate constant (Figure 5A in [6] and see Figure 1C above) to a macromolecular crowding-driven transition between two kinetic states (Scheme 1 in [6]). This transition could be accompanied by a 2D condensation phase transition of DnaA on the membrane surface, in which the dilute phase consists of proteins in state II and the condensed phase is composed of proteins residing in state I. Although we do not discuss here the details of the attractive interaction leading to this transition, we wish to note that similar transitions have been previously observed and theoretically suggested [37]. Such a theoretical approach may be used for describing DnaA behaviour on the membrane. We denote by *θ* the occupancy of the membrane surface by the protein, with 0 *≤ θ ≤* 1 (*θ*=1 corresponds to close packing of the proteins) and assume that the system is below its critical temperature for the phase transition. Accordingly, we may expect three regimes: *θ* < *θ*_d_, in which there is a single dilute phase, *θ* > *θ*_c_, in which there is a single condensed phase and *θ*_d_ < *θ* < *θ*_c_, where two phases coexist: dilute phase with occupancy *θ*_d_ and a condensed phase with occupancy *θ*_c_.

**Figure 5:**
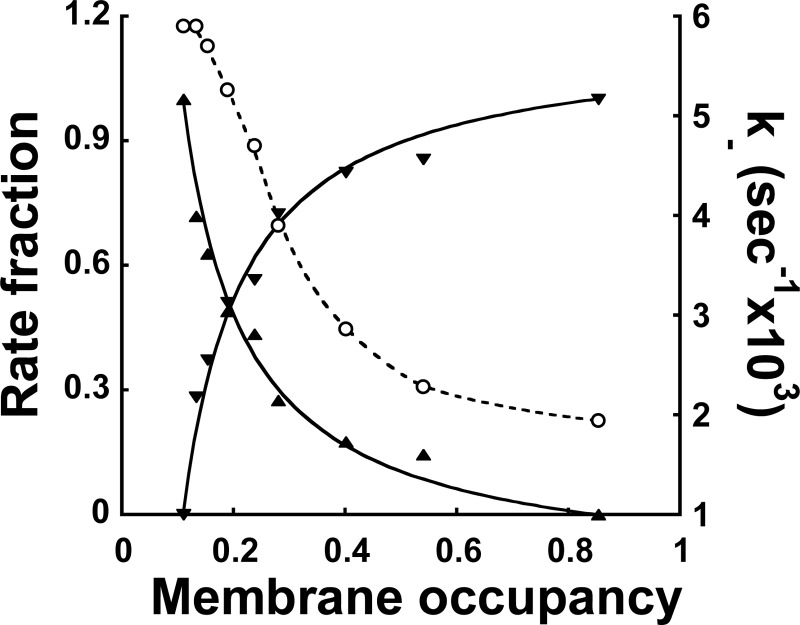
Comparison of experimental MANT–ATP·DnaA dissociation kinetics with the phase transition model prediction The dissociation kinetics measured at different membrane occupancies as described in [6] (see Figure 4 therein) were fitted either with a single exponential decay function resulting in a varying dissociation rate constant *k*- (○, connected with a dashed line just to guide the eye) or with a double exponential decay function (eqn 4) using fixed extreme rate constant values *k*_d_ and *k*_c_ and the exponents' fractions as free fit parameters. The obtained values of *f*_d_ (▲) and *f*_c_ (▼) are plotted against membrane occupancy. One experimental series (of seven averaged in Figure 1C) was used for this alternative fitting. For a comparison with the model, theoretical values of *f*_d_ and *f*_c_ calculated from eqn 5 are presented (continuous lines). See the text for details.

Consider now the kinetics of the nt–DnaA dissociation. In the single-phase regions, namely, dilute (*θ* < *θ*_d_) and condensed (*θ* > *θ*_c_), we may expect first-order kinetics with dissociation rate constants *k*_d_ and *k*_c_ respectively. Our interpretation is also based on the assumption that, due to various motional constraints affecting nt affinity, the reactivity of the condensed phase is markedly lower than that of the dilute phase, with *k*_d_ > *k*_c_. There could also be a weak dependence on *θ* in the single-phase regions, *θ* < *θ*_d_ and *θ* > *θ*_c_, but in order to simplify the discussion, we ignored this possibility.

Thus, the occupancy of the DnaA–nt complex θ*(t) is given by θ*(*t*)=θ*(0)*e*^− *k*_d_*t*^ in the dilute phase and θ*(*t*)=θ*(0)*e*^− *k*_c_*t*^ in the condensed phase.

Let us now consider the kinetics in the two-phase coexistence region, *θ*_d_ < *θ* < *θ*_c_. In each phase the kinetics will continue to be first order, so that the overall decay will be the sum of the two contributions, leading to the sum of two exponentials with proper weight given to each phase proportional to its abundance. Thus, the overall complex occupancy is as follows:

$$\theta^{*}\left(t\right)=x_{d}\theta_{d}^{*}\left(0\right)e^{-k_{d}t}+x_{c}\theta_{c}^{*}\left(0\right)e^{-k_{c}t}$$

Where *x*_d_=(*θ*_c_ − *θ*)/(*θ*_c_ − *θ*_d_) and *x*_c_=(*θ* − *θ*_d_)/(*θ*_c_ − *θ*_d_) are the fractions of the area that each of the phases occupy. If we assume that initially all the DnaA proteins on the surface contain bound nt, we have in the two-phase region θ*_d_ *(0)=θ*_d_, θ*_c_ *(0)=θ*_d_ and θ*(0)=θ.

Consequently, if the experimental dissociation data are fitted with a double exponential decay, such that

4 $$\frac{\theta^{*}\left(t\right)}{\theta }=f_{d}e^{-k_{d}t}+f_{c}e^{-k_{c}t}$$

with *f*_d_ + *f*_c_=1, then the theoretically predicted fractions of the fast (*f*_d_) and slow (*f*_c_) components of the kinetics are

5 $$f_{d}=\frac{x_{d}\theta_{d}}{\theta }\;\;\mathrm{and}\;\;f_{c}=\frac{x_{c}\theta_{c}}{\theta }$$

Fitting the MANT–ATP·DnaA dissociation kinetics within the ‘two-phase’ region with a double exponential decay function, using *k*_d_=5.9 × 10^−3^ and *k*_c_=2.0 × 10^−3^ s^−1^ from the ‘single-phase’ decay curves [6] and taking *f*_d_ and *f*_c_ as free fit parameters allows one to compare our experiment with this theoretical model. In Figure 5, the theoretical values of *f*_d_ and *f*_c_ (eqn 5), with the choice of parameters *θ*_d_=0.11, *θ*_c_=0.85 respectively, are compared with the experimental values. The theory appears to predict the dominant trend in the observed behaviour, even if there are some deviations from the theoretical line.

Interestingly, by adopting the mean-field approximation for the lattice-gas model on a square lattice in 2D, the (upper) critical temperature is related to the interaction energy *w* between nearest-neighbour proteins by *k*_B_*T*_c_=*w*. Furthermore, the well-known theoretical shape of the (mean-field) coexistence curve ([38] Chapter 8),

$$\frac{T}{T_{c}}=\frac{2(2\theta -1)}{\ln\left(\frac{\theta }{1-\theta }\right)}$$

shows that, for *θ*_d_ ≅ 0.11, the temperature is *T* ≅ 0.75 × *T*_c_, implying *w* ≅ 1.5 × *k*_B_*T*. Thus, the non-vanishing value of *θ*_d_ suggests that the effective attractive interaction between proteins is rather weak, i.e. within 1–2 *k*_B_*Ts*. Note also that *θ*_d_ ≅ 1 − *θ*_c_, implying that the phase diagram is roughly symmetric around 
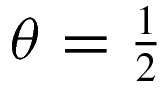
 and that the main interactions are pairwise.

To summarize, the above model when applied to the previous kinetic measurements enabled us to conclude that DnaA activation on the membrane occurs via a transition between two distinct states of the protein accompanied by a condensation transition.

## DISCUSSION

The membrane-mediated pathway of DnaA reactivation has gained support mainly from findings indicating the membrane's need for *in vitro* DNA synthesis [7–9], studies of DnaA binding to model membranes [6,33,34,39,40] and *in vivo* functional effects of phospholipid synthesis mutations [41–43]. We have previously reported two kinetically distinct forms of DnaA bound to the membrane, a co-operative transition between which may serve as an activation switch for initiating DNA replication [6]. Previously, we have suggested a molecular model for DnaA–membrane binding based on *in vivo* membrane localization of DnaA fragments [13]. However, the molecular mechanism underlying the on-membrane activation of the nt exchange remains obscure. Previously it was shown that two states of DnaA on the membrane are macromolecular crowding dependent [6], suggesting oligomerization of the protein [14,15]. It is well known that the N-terminal domain of the protein is essential for DnaA oligomerization in solution and specifically on *oriC* [18–21,29]. In the present study, we explored whether oligomerization in general and the N-terminal in particular are important for DnaA rejuvenation by the membrane.

The finding that tDnaA loses the capability for co-operative transformation between states I and II on the membrane (Figure 1) indicates that the N-terminal is indispensable in this phenomenon as well. Moreover, based on the thermodynamic characteristics (Figure 1D; Table 1), it can be concluded that at high tDnaA density on the membrane surface and in the aqueous phase, the dissociation rate constant is similar to that of wtDnaA and that it probably corresponds to the same state I of the protein. However, the state of tDnaA at low membrane occupancy is dramatically different from that of the wild-type as well as from its own state at high membrane occupancy. The distinct tDnaA 'state II' apparently exhibits the macromolecular crowding effect, yet with no kinetic expression at physiological temperatures. In the absence of the N-terminal, the interaction between tDnaA monomers is still possible through another DnaA–DnaA binding site (α-10 helix), which is retained after truncation [33]. This result indicates that the N-terminal of DnaA is responsible for the formation of active state II.

The transition from state II to I of wtDnaA was accompanied by spectral changes in the environmentally sensitive fluorophore MIANS (Figure 3) specifically bound to N-terminal cysteines (Figure 2). The 2-fold increase in the fluorescence intensity at state I is apparently a manifestation of a major structural transformation in the N-terminal region.

Cross-linking experiments have revealed that states I and II correspond to two distinct oligomeric forms of DnaA. The DnaA trimerization occurs co-operatively in the same range of the lipid:DnaA ratio as the changes in the nt dissociation rate constant (Figure 4C), indicating that state II of the protein on the membrane corresponds to a trimeric or higher form of DnaA. Moreover, the orientations of DnaA molecules relative to each other are different between the states, which follow from the existence of three separately running dimers (Figures 4B and 4D). The D3 dimer represents the orientation of protein molecules in oligomers on the membrane at state II. Paradoxically, the transition to state II occurs at low protein density on the membrane, hinting at a sparse functional oligomeric configuration, rather than a dense aggregation, which could be expected according to the effects of surface macromolecular crowding [14,15].

The relatively low amount of cross-linking products in our experiments (up to 20% overall) is due to the low concentration of the cross-linker used and the apparent probability dependence of double, triple and higher cross-links. However, it should be taken into account that the existence of larger oligomeric forms, represented by the trimer and the less visible tetramer (Figure 4B), is also possible, but it was hardly detectable due to the low cross-linking probability. Moreover, the detected dimers and trimers most probably are just parts of the higher order oligomers.

The transition between states I and II can be plausibly explained by a 2D condensation phase transition model (Figure 5). This model strengthens the notion of the existence of two states of DnaA on the membrane and points to the possibility that the switch between the two states can be much sharper than that experimentally observed. This suggests its high physiological significance in DnaA activation. Consequently, we would like to comment on the possibility of the membrane protein density serving as an on-off switch *in vivo*. The ability to have access to the two-phase region in our *in vitro* experiments is associated with the fact that the ratio of protein to lipid is given. This is equivalent to the liquid–gas two-phase coexistence that is accessible at a fixed molar volume [38]. Yet the situation *in vivo* is probably different. It has been suggested that *E. coli* cells contain a large reservoir of the chromosome-bound DnaA [44,45]. Assuming that the chemical potential of DnaA in this reservoir is controlled by cellular regulatory systems and that it therefore determines the chemical potential in the cytoplasm and on the membrane surface (assuming thermodynamic equilibrium), a tiny change in the DnaA chemical potential around the phase transition value will induce a full transition from one phase to the other on the membrane surface. This is equivalent to observing the liquid–gas phase transition at constant pressure rather than at constant molar volume. For example, if the chemical potential changes from a value slightly smaller than the critical value to a slightly larger value, the membrane surface will be fully transformed from the diluted phase to the condensed phase. Since the two phases are associated with different dissociation constants, *k*_d_ and *k*_c_, the result is a switch between the two of them.

The transformation from kinetic state I to state II may be described by this simplified model for transitions between the condensed and diluted phases of the protein on the membrane surface. Combining this model with the above described results, we suggest the following scheme, which illustrates the conformational transformation and the oligomerization states of DnaA upon binding to the membrane at high and low macromolecular surface density (crowding).

Both wild-type and tDnaA assume a similar monomeric conformation upon membrane binding, distinct from that in the water phase (spheres to cubes in Scheme 1, used to symbolize the structure). At high surface density, both are driven into a condensed phase (square prisms) with protein–protein interactions overwhelming the lipid–protein interactions, leading to similar conformations characterized by a slow nt exchange, state I. At low DnaA surface density, the higher orientation freedom enables the wtDnaA to assemble into a quaternary structure in which the N-terminals connect the monomers, as proposed by Berger and colleagues [23]. The resulting oligomers are characterized by short-range cross-linking (Figure 4), with monomer units presumably adapting a different conformation (denoted by a cylinder); they possess distinct kinetic properties such as a fast nt exchange, state II (Table 1). Noteworthy, the intermediate monomeric form of wtDnaA (denoted by a cube) is proposed based on the properties detected only for the tDnaA in the dilute phase (Table 1 and the upper part of Scheme 1). In addition, the monomer–oligomer conformational transformation (cubes-to-cylinder) is presumably driven by excessive lipid–protein interactions in the dilute phase, replacing the mainly protein–protein interactions in the condensed phase. We have previously suggested the mode of specific membrane binding of DnaA through hydrophobic continuity on domain III of the protein, leading to its conformational change [13]. In the present study, we extend the model by suggesting that the mechanism underlying the membrane rejuvenation of native DnaA is associated with its density-driven co-operative oligomerization.

## Abbreviations

AAA+: ATPases associated with a variety of cellular activities
BMOE: 1.2-bis-maleimidoethane
MANT-ATP: 2′-(or-3′)-O-(N-Methylanthraniloyl) Adenosine 5′-Triphosphate
MIANS: 2-(4-maleimidoanilino)naphthalene-6-sulfonic acid
NEM: N-ethylmaleimide
NTA: nitrilotriacetic acid
PL:Pr: phospholipid-to-protein ratio
SOPG: 1-Stearoyl-2-oleoyl-*sn*-glycero-3-[phospho-*rac*-(1-glycerol)]
tDNA: truncated DnaA
wtDnaA: full-length DnaA

## References

[1] Boye E., Lobner-Olesen A., Skarstad K. (2000). Limiting DNA replication to once and only once. EMBO Rep..

[2] Lee D.G., Bell S.P. (2000). ATPase switches controlling DNA replication initiation. Curr. Opin. Cell Biol..

[3] Sekimizu K., Bramhill D., Kornberg A. (1987). ATP activates dnaA protein in initiating replication of plasmids bearing the origin of the *E. coli* chromosome. Cell.

[4] Katayama T., Kubota T., Kurokawa K., Crooke E., Sekimizu K. (1998). The initiator function of DnaA protein is negatively regulated by the sliding clamp of the *E. coli* chromosomal replicase. Cell.

[5] Kaguni J.M. (2006). DnaA: controlling the initiation of bacterial DNA replication and more. Annu. Rev. Microbiol..

[6] Aranovich A., Gdalevsky G.Y., Cohen-Luria R., Fishov I., Parola A.H. (2006). Membrane-catalyzed nucleotide exchange on DnaA. Effect of surface molecular crowding. J. Biol. Chem..

[7] Sekimizu K., Kornberg A. (1988). Cardiolipin activation of dnaA protein, the initiation protein of replication in *Escherichia coli*. J. Biol. Chem..

[8] Castuma C.E., Crooke E., Kornberg A. (1993). Fluid membranes with acidic domains activate DnaA, the initiator protein of replication in *Escherichia coli*. J. Biol. Chem..

[9] Crooke E., Castuma C.E., Kornberg A. (1992). The chromosome origin of *Escherichia coli* stabilizes DnaA protein during rejuvenation by phospholipids. J. Biol. Chem..

[10] Saxena R., Fingland N., Patil D., Sharma A.K., Crooke E. (2013). Crosstalk between DnaA Protein, the Initiator of *Escherichia coli* chromosomal replication, and acidic phospholipids present in bacterial membranes. Int. J. Mol. Sci..

[11] Fujimitsu K., Senriuchi T., Katayama T. (2009). Specific genomic sequences of *E. coli* promote replicational initiation by directly reactivating ADP-DnaA. Genes Dev..

[12] Leonard A.C., Grimwade J.E. (2009). Initiating chromosome replication in *E. coli*: it makes sense to recycle. Genes Dev..

[13] Regev T., Myers N., Zarivach R., Fishov I. (2012). Association of the chromosome replication initiator DnaA with the *Escherichia coli* inner membrane *in vivo*: quantity and mode of binding. PLoS One.

[14] Minton A.P. (1999). Adsorption of globular proteins on locally planar surfaces. II. Models for the effect of multiple adsorbate conformations on adsorption equilibria and kinetics. Biophys. J..

[15] Minton A.P. (2000). Effects of excluded surface area and adsorbate clustering on surface adsorption of proteins I. Equilibrium models. Biophys. Chem..

[16] Crooke E., Thresher R., Hwang D.S., Griffith J., Kornberg A. (1993). Replicatively active complexes of DnaA protein and the *Escherichia coli* chromosomal origin observed in the electron microscope. J. Mol. Biol..

[17] Messer W., Blaesing F., Jakimowicz D., Krause M., Majka J., Nardmann J., Schaper S., Seitz H., Speck C., Weigel C. (2001). Bacterial replication initiator DnaA. Rules for DnaA binding and roles of DnaA in origin unwinding and helicase loading. Biochimie.

[18] Weigel C., Schmidt A., Seitz H., Tungler D., Welzeck M., Messer W. (1999). The N-terminus promotes oligomerization of the *Escherichia coli* initiator protein DnaA. Mol. Microbiol..

[19] Felczak M. M., Simmons L. A., Kaguni J. M. (2005). An essential tryptophan of *Escherichia coli* DnaA protein functions in oligomerization at the *E. coli* replication origin. J. Biol. Chem..

[20] Simmons L.A., Felczak M., Kaguni J.M. (2003). DnaA Protein of *Escherichia coli*: oligomerization at the *E. coli* chromosomal origin is required for initiation and involves specific N-terminal amino acids. Mol. Microbiol..

[21] Abe Y., Jo T., Matsuda Y., Matsunaga C., Katayama T., Ueda T. (2007). Structure and function of DnaA N-terminal domains: specific sites and mechanisms in inter-DnaA interaction and in DnaB helicase loading on oriC. J. Biol. Chem..

[22] Erzberger J.P., Mott M.L., Berger J.M. (2006). Structural basis for ATP-dependent DnaA assembly and replication-origin remodeling. Nat. Struct. Mol. Biol..

[23] Erzberger J.P., Pirruccello M.M., Berger J.M. (2002). The structure of bacterial DnaA: implications for general mechanisms underlying DNA replication initiation. EMBO J..

[24] Ogura T., Wilkinson A.J. (2001). AAA+ superfamily ATPases: common structure-diverse function. Genes Cells.

[25] Clarey M.G., Erzberger J.P., Grob P., Leschziner A.E., Berger J.M., Nogales E., Botchan M. (2006). Nucleotide-dependent conformational changes in the DnaA-like core of the origin recognition complex. Nat. Struct. Mol. Biol..

[26] Yao N., Coryell L., Zhang D., Georgescu R.E., Finkelstein J., Coman M.M., Hingorani M.M., O'Donnell M. (2003). Replication factor C clamp loader subunit arrangement within the circular pentamer and its attachment points to proliferating cell nuclear antigen. J. Biol. Chem..

[27] Akoev V., Gogol E.P., Barnett M.E., Zolkiewski M. (2004). Nucleotide-induced switch in oligomerization of the AAA+ ATPase ClpB. Protein Sci..

[28] Felczak M.M., Kaguni J.M. (2004). The box VII motif of *Escherichia coli* DnaA protein is required for DnaA oligomerization at the *E. coli* replication origin. J. Biol. Chem..

[29] Scholefield G., Errington J., Murray H. (2012). Soj/ParA stalls DNA replication by inhibiting helix formation of the initiator protein DnaA. EMBO J..

[30] Bradford M.M. (1976). A rapid and sensitive method for the quantitation of microgram quantities of protein utilizing the principle of protein-dye binding. Anal. Biochem..

[31] Laemmli U.K. (1970). Cleavage of structural proteins during the assembly of the head of bacteriophage T4. Nature.

[32] Bornstein P., Balian G. (1977). Cleavage at Asn-Gly bonds with hydroxylamine. Methods Enzymol..

[33] Garner J., Crooke E. (1996). Membrane regulation of the chromosomal replication activity of *E. coli* DnaA requires a discrete site on the protein. EMBO J..

[34] Aranovich A., Parola A.H., Fishov I. (2007). The reactivation of DnaA(L366K) requires less acidic phospholipids supporting their role in the initiation of chromosome replication in *Escherichia coli*. FEBS Lett..

[35] Hawe A., Sutter M., Jiskoot W. (2008). Extrinsic fluorescent dyes as tools for protein characterization. Pharm. Res..

[36] Gupte S.S., Lane L.K. (1979). Reaction of purified (Na,K)-ATPase with the fluorescent sulfhydryl probe 2-(4'-maleimidylanilino)naphthalene 6-sulfonic acid. Characterization and the effects of ligands. J. Biol. Chem..

[37] Mbamala E.C., Ben-Shaul A., May S. (2005). Domain formation induced by the adsorption of charged proteins on mixed lipid membranes. Biophys. J..

[38] Atkins P.W., Paula J.D. (2001). Physical Chemistry.

[39] Garner J., Durrer P., Kitchen J., Brunner J., Crooke E. (1998). Membrane-mediated release of nucleotide from an initiator of chromosomal replication, *Escherichia coli* DnaA, occurs with insertion of a distinct region of the protein into the lipid bilayer. J. Biol. Chem..

[40] Kitchen J.L., Li Z., Crooke E. (1999). Electrostatic interactions during acidic phospholipid reactivation of DnaA protein, the Escherichia coli initiator of chromosomal replication. Biochemistry.

[41] Xia W., Dowhan W. (1995). *In vivo* evidence for the involvement of anionic phospholipids in initiation of DNA replication in *Escherichia coli*. Proc. Natl. Acad. Sci. U.S.A..

[42] Zheng W., Li Z., Skarstad K., Crooke E. (2001). Mutations in DnaA protein suppress the growth arrest of acidic phospholipid-deficient *Escherichia coli* cells. EMBO J..

[43] Fingland N., Flatten I., Downey C.D., Fossum-Raunehaug S., Skarstad K., Crooke E. (2012). Depletion of acidic phospholipids influences chromosomal replication in *Escherichia coli*. Microbiologyopen.

[44] Kitagawa R., Mitsuki H., Okazaki T., Ogawa T. (1996). A novel DnaA protein-binding site at 94.7 min on the *Escherichia coli* chromosome. Mol. Microbiol..

[45] Roth A., Messer W. (1998). High-affinity binding sites for the initiator protein DnaA on the chromosome of *Escherichia coli*. Mol. Microbiol..

